# Moving beyond normative philosophies and policy concerns: a sociological account of place-based solidarities in diversity

**DOI:** 10.1186/s40878-018-0083-5

**Published:** 2018-05-17

**Authors:** Stijn Oosterlynck

**Affiliations:** Research Centre on Inequality, Poverty, Social Exclusion and the City (OASeS) & Antwerp Urban Studies Institute, University of Antwerp, Antwerp, Belgium

**Keywords:** Solidarity, Super-diversity, Social transformation, Place, Sociology

## Abstract

In this commentary, I think with and beyond the normative philosophies and policy-oriented frameworks on how to deal with diversity in contemporary societies formulated by Zapata-Barrero and Modood. I propose to integrate elements of both perspectives in a empirically grounded sociological account of how place-based solidarities in diversity are nurtured in everyday life. Although there is much to be recommended about the arguments of Modood and Zapata-Barrero, I argue that what is needed is an analytical framework that does not a priori privilege specific sources of solidarity on normative-philosophical or policy grounds. We need to focus instead on how people mobilise different sources of solidarity in their attempts to take shared responsibility for the concrete places where they live, work, learn and play together in superdiversity. This micro-level focus does not mean that one ignores macro-level processes. Also, more attention should be paid to the transformative nature of solidarities in diversity.

## Introduction

In this commentary I reflect on the possibility of solidarity in diversity, both in conceptual and empirical terms, by thinking with and beyond Zapata-Barrero and Modood (Modood, 2017; Zapata-Barrero, 2017). My aim is not so much to choose sides in the debate between multiculturalism and interculturalism, but to integrate elements of both perspectives in an analytical framework that does not a priori – on political-philosophical or policy grounds – privilege one source of solidarity in diversity over another and looks at their articulation in concrete empirical cases. Although normative and policy-based arguments are valuable and often draw extensively on concrete experiences, we need to move beyond these and engage in on-the-ground empirical analysis of how new forms of solidarity in diversity are nurtured in everyday life. What I propose here is not an empiri*cist* analysis devoid of conceptual thinking, but a sociological approach of living in diversity in contemporary society as a ‘social fact’ to be analysed empirically (see Kivisto, 2012 for a similar argument). I will draw on classical sociological theory to distinguish four sources of solidarity, which I argue are more adequately capturing the complex, multiple and overlapping sources of actually existing solidarities in diversity. I will illustrate this argument with stylized examples from a large-scale collaborative research project on innovative forms of solidarity in diversity.^1^

## Place-based solidarities in diversity

In his contribution Zapata-Barrero (2017) points out the very different ‘origins in expertise’ of the multicultural and interculturalist paradigm. According to him, multiculturalism originates from the academic world, while interculturalism has its roots in policy-making. This makes interculturalism more evidence-based than multiculturalism whose legitimacy problems stem from its “empirical shortcomings” (p. 11). Whether true or not, both multiculturalism and interculturalism seem to propose a prescriptive rather than an analytical framework. Multiculturalism is a rights-based ‘political theory’ that according to Modood is “centred on equal citizenship” and that “cherishes historical identities – minority and majority – and embraces what some might call conservative institutions […] but insists on their extension or adaptation to include ethno-religious groups and identities” (Modood, 2017, p. 3). Interculturalism, as defined by Zapata-Barrero, is a ‘policy philosophy’ (p.16) that stresses the importance of dialogue – a focus also forcefully claimed by Modood in his defence of multiculturalism – and does so by referring to the empirical research on Allport’s contact hypothesis. From an interculturalist perspective, dialogue across cultural differences requires ‘national civic policy’ to create ‘unity’, stress the ‘duties’ of immigrants and develop the conditions to make social contact and dialogue between diverse citizens possible. Zapata-Barrero justifies this on the ground of the ‘backlash against multiculturalism’ and the political rise of anti-immigrant populism. In this context, he argues for a focus on “common bonds rather than differences” (p. 3), recognizing diversity as “an advantage and a resource” and identifies “community cohesion and a diversity-based common public culture” as its main “normative policy drivers”.

Both paradigms contain highly relevant insights for an analytical framework on living in diversity and I will discuss these insights in more detail below. Still, as both paradigms gravitate towards prescription – whether based on normative philosophy (multiculturalism) or grounded in policy concerns (interculturalism) – they tend to work with a priori (i.e. posited before empirical analysis) assumptions on what makes living in diversity possible. For an adequate empirical analysis of living in diversity as a social fact, a broader analytical framework is needed. In this contribution I will discuss what I consider the most relevant insights from the articles of Modood (2017) and Zapatta-Barrero (2017) within the context of an analytical framework on new forms of solidarity in diversity that I – together with colleagues from a variety of disciplines, most notably educational science, human geography, sociology and political science – have been developing (see Oosterlynck, Loopmans, Schuermans, Van den Abeele, & Zemni, 2016 for a full account of this analytical framework). We argue that – at least in Europe – solidarity has been thoroughly ‘nationalised’ in the course of the twentieth century. In a review essay on the politics of solidarity, Van Kersbergen writes that “the origins of modern social policy were found in the nation- and state-building efforts of the etatist elites who used the granting of social rights to enhance the integration of society” (Van Kersbergen, 2006, p. 379). Stjerno explains how “after World War I, the *nation* became the frame of reference for solidarity, and after World War II, the national welfare state was established and gradually legitimised by a language of solidarity” (Stjerno, 2004, p. 342). Solidarity has thus become pre-dominantly organised within territorialised welfare states and operated by centralised and largely secularised welfare bureaucracies. This means that the legitimacy of national welfare-state based forms of solidarity are – due to being tied up with nation-state formation – strongly predicated on the *perceived* cultural homogeneity of nation-state populations, which is nurtured through intergenerational transfers of historical narratives, norms and values within relatively stable national political communities.

This is particularly the case in Europe, which has for long been reluctant to conceive itself as immigration society (Mollenkopf & Hochschild, 2010). Since increasing ethnic and cultural diversity interrupts the intergenerational continuity in nation-states, it threatens to erode the basis on which the popular legitimacy of national solidarities rest. As Kymlicka (2015) recently argued: multicultural policies that aim to recognise migration-related cultural differences weaken “bonds of nationhood and hence the ability to secure stability and solidarity” (p. 6), thus giving rise to “welfare chauvinism” or what he calls “solidarity without inclusion”. Based on the extensive empirical analysis of 21 case studies of solidarity in diversity in the Flemish Region and Brussels (Belgium), we observe that new forms of solidarity *in diversity* shift the spatio-temporal framework through which solidarities are generated away from territorialised nation-states and intergenerational continuity to *places*, in a relational rather than territorial sense (Amin, 2004; Massey, 2004), and the concrete *practices* of diverse individuals and ‘groupings’ (cfr. Modood) in these places. In the ‘here’ and ‘now’, solidarities in diversity come into being when joint responsibility is taken for the urban places such as schools, workplaces, leisure areas and residential areas that people share in diversity.

This observation has strong affinities with Zapata-Barrero’s (2017) formulation of interculturalism as focused on proximity – *the* defining feature of place as a principle of socio-spatial structuration (Jessop, Brenner, & Jones, 2008) – and practice. Zapata-Barrero frames his local and pragmatist perspective on living in diversity as “a *policy rebellion of cities* against the state domination of policy in recent decades” (p.13), with the latter referring to “the state-centred and universalist view of multicultural policies” (p.3). While I endorse his focus on urban and public space as privileged spheres of action for generating solidarity in diversity, a focus which is not only central to the aforementioned research but to most empirical studies on superdiversity and conviviality (Jones et al., 2015; Oosterlynck, Schuermans, & Loopmans, 2017; Wessendorf, 2013), I see no analytical reason to limit action in this context to policies (“the need for a policy the main target of which is to encourage contact among people”, Zapata-Barrero, 2017, p. 17) as citizen-based actions and interventions equally carry the potential of nurturing solidarity in diversity. Indeed, in our case study-based research in the Flemish and Brussels regions civil society professionals – whether paid or volunteers – seem to be central actors, while many policy makers seem pre-occupied with establishing what Zapata-Barrero calls ‘national civic policies’.

## Sources of solidarity

Related to this is the observation that policy-makers tend to focus on only two sources of solidarity, namely contact (or encounter) and shared norms and value. Although Zapata-Barrero is certainly right in stressing the value of contact (under particular circumstances) in generating solidarity in diversity, our empirical analysis of micro-level interactions in particular places makes visible a wider set of sources of solidarity. While norms and values, and encounter are central to the multiculturalism and interculturalism debate, our social theory informed empirical research has shown that interdependency and struggle are equally powerful, but all too often ignored sources of solidarity, both in normative philosophical accounts on governing diversity and in diversity policy-making. By a priori privileging norms and values and/or encounter over other sources of solidarity, Zapata-Barrero (2017), through his focus on national civic culture, tends to frame social cohesion in superdiverse societies too narrowly in communitarian terms or as Amin puts it “common life based on reduced or reconciled differences and strengthened social and community ties” (Amin, 2012, p. 3). The social is, however, more than ‘collective identification’ and ‘dialogue’. Surely, intercultural contact is a crucial aspect in solidarity generating processes, but it is “not [a] sufficient condition for multicultural understanding” (Amin, 2002, p. 969).

The notions of interdependency and struggle as important sources of solidarity in modern societies have a long lineage in sociological theory. Durkheim famously distinguished mechanical and organic forms of solidarity, with the latter referring to the type of solidarity that emerges in industrialised societies with extensive divisions of labour (Durkheim, 1893/1984). Interdependency does not only refer to the division of labour in capitalist economies, but can also refer to the purposeful creation of interdependencies in common projects. Amin, for example, argues that “a politics of encounter requires active intermediation by third parties, managed interaction or common projects in order to undo settled behaviour, build interdependence or common purpose, catalyse positive feelings” (Amin, 2010, p. 3). It requires ‘contact spaces’ to be “structured as spaces of interdependence” (Amin, 2002, p. 969) by setting up common projects in which citizens need each other to reach a common goal. This is illustrated in a case study on the post order company Spring Global Mail in the Belgian city of Mechelen, in which around 50 employees from a variety of ethnic-cultural backgrounds process mail orders in a fixed workflow (Spijkers & Moris, 2014 – DieGem case study 1).^2^ Through daily cooperation small practices of solidarity across cultural lines develop. Employees learn each other how to best carry out work tasks and provide emotional support to each other on difficult days. On this work floor, efficiency trumps concerns about cultural differences. Due to time pressure (‘All post must be out in the evening’), interactions on the work floor are mainly focused on getting the work flow going.

Common projects do not have to be in the sphere of the market economy with its strong pressure towards efficiency and a pragmatic attitude. In a case study of an informal refugee camp in the Maximiliaan park in Brussels, we observed how the refugee camp became a joint project of a range of volunteers from very different cultural and socio-economic backgrounds (Depraetere, Oosterlynck & Vandenabeele, 2017 – DieGem case study 18).^3^ Whereas the refugee crisis triggered humanitarian responses in the wider population (‘we have to do something’), the refugee camp offered a concrete space for the channelling and organisation of emerging practices of solidarity. An informal ‘division of labour’ emerged in which volunteers took up tasks such as providing hot and cold meals, keeping an eye on the safety in the camp, receiving donations of food, blankets and cloths and managing the stock. Some volunteered to act as coordinators of this informal division of labour, which, although it frequently led to tensions, organised the many practices of solidarity in diversity.

Social struggle is another important, but often ignored source of solidarity. The lineage of struggle as a source of solidarity can be traced back to the work of Karl Marx and Max Weber (Stjerno, 2004). Since the scarce resources in society are unevenly distributed, some social groupings are misrecognised and not (well) represented in decision-making processes, social struggles to address these are an endemic feature of each society. The pooling of resources and joining of forces against a shared enemy, is – as Marx described for social classes and Weber also for status groups and ‘political parties’ – a powerful, yet exclusionary source of solidarity. Although increasing ethnic and cultural diversity have made the terrain on which these struggles are waged much more complex (Fraser, 1995), we observed that place-based struggles are an important catalyst to generate solidarities in diversity. One example from our case studies is the collective housing project *Leeggoed* in the Brussels municipality of Elsene (Depraetere, Vandenabeele & Oosterlynck, 2015 – DieGem case study 11).^4^ *Leeggoed* was realised by a diverse group of citizens who lived in precarious housing situations and joined together as a group to realise their right to housing with the support of several social organisations. As the search for housing was not successful, they decided to occupy two empty social housing units and used this as leverage to enter into negotiations with the social housing corporation. In the end the social housing corporation gave them access to four apartments, which the prospective residents then renovated themselves. During the years of struggle and the renovation works, strong bonds of solidarity were forged. Within the collective housing project, residents do not share everything and there are regularly small and big conflicts, but they have developed family-like relationships by living together, negotiating the rules and future of the housing project and organising neighbourhood activities.

However, if we want to move beyond the a priori privileging of certain sources of solidarity, we need to analyse concrete instances of living together in diversity from the perspective of how different sources of solidarity are combined and articulated in specific contexts. Another case study of practices of solidarity in diversity in the work place offers a good example (Debruyne, 2015 – DieGem case study 5).^5^ Tower Automotive is a car assemblage factory in Ghent and has a superdiverse workforce, with employees of more than 40 different nationalities. Just as in the aforementioned case of Spring Global Mail, the pressures of the production process push towards pragmatic cooperation across cultural lines. But this is further supported by on the one hand a company policy that explicitly celebrates diversity as something positive (reflected in the slogan ‘Tower promotes diversity by providing equal opportunities to all’) and that pursues a strict anti-discrimination policy on the factory floor and on the other hand the ‘proximity politics’ of trade union representatives on the work floor (e.g. attending funerals of family members of employees, installing someone’s printer, helping people to make arrangements to resettle debts). Setting pro-diversity norms and values and encountering and supporting others as fellow human beings and not just colleagues from work leads to a workplace on which solidarity in diversity is generated.

## Socially stratified places

Focusing on what Amin has called “everyday lived experiences and local negotiations of difference” (Amin, 2002, p. 967) in urban schools, work and leisure places and residential environments where citizens of diverse origins are (having) to live together, does not mean that the macro-level processes of communication between cultural groupings that according to Modood are lacking in Zapata-Barrero’s Interculturalism are not important. Modood is certainly right in warning that a micro-level focus should not lead us to neglect macro-level processes and the reality of groupings. However, Modood limits his criticism to macro-level *dialogue* (and presumably diversity policies), while a macro-level focus should also look at other society-wide processes, for example the workings of national welfare regimes or processes of economic (re)structuring. This can be addressed by approaching urban places as socially produced and hence structured through wider societal processes of stratification, particular histories and spatial arrangements. Valentine warns against romanticising micro-level encounters: “encounters never take place in a space free from history, material conditions, and power” (see also Featherstone in Oosterlynck et al., 2017; Valentine, 2008, p. 333). When diverse citizens find themselves thrown together in particular places, encounters that happen there take place on an uneven terrain. Take for example the case study of HIVSET, a superdiverse secondary school in the provincial town of Turnhout (Schuermans, 2016 – DieGem case study 17).^6^ In recent years, the school has been confronted with a sudden surge of migrant newcomers, which were taking intensive Dutch language training (separate from the other pupils). In response to a number of racist incidents, the school started using the breaks to forge connections between the newcomers and established school population by organising joint activities (e.g. sports, knitting workshops). Despite attempts to forge connections between the group of migrant newcomers and the established school population and accommodate cultural differences (e.g. allowing the wearing of a headscarf), the approach of the school was to use social mixing strategies to socialise newcomers in the (perceived) Belgian norms and values and conceive of interaction as taking place between ‘a majority’ and ‘a minority’. Power also functions in the scalar structuration of places (i.e. the hierarchical organisation of the relations between different spaces) (Brenner, 2001), which has important effects on the degree to which place-based solidarities in diversity can travel beyond the specific places where they are nurtured. For example, in the aforementioned case of Tower Automotive, there clearly is an asymmetrical relation between this subcontracting firm and its superdiverse workforce on the one hand and the contracting car company Volvo with its better paid and educated and more white workforce on the other hand. Hence, the place-based solidarities nurtured in and around the work floor of Tower Automotive did not lead to the challenging of the socio-economic inequalities, which very much run along ethnic lines, in the supply chain in the local car industry. From that perspective, I endorse Modood’s alternative interpretation of the anti-essentialist consensus on group identities, namely that groups – or *groupings* as he calls them aptly – are not just subject to ‘ascriptive Othering’ (p.8), but have an ‘existence of its own’. Although their concrete existence should be analysed empirically in every particular superdiverse context, in this case (as in many others), we find clear evidence that encountering diversity in specific places is not solely a matter of individuals, but also of cultural groupings. In this sense, Modood is right to stress the “normative significance of recognising groups in addition to individual citizens” (p.4).

My final point revolves around the observation from our case studies that place-based solidarities in diversity very often are transformative in nature. This transformative character of solidarity in diversity does not sit easily with a long tradition to think solidarity in the context of a search for social *order* (see e.g. Lockwood, 1992; Turner, 1998). As Stjerno writes “the idea of solidarity was part of a wider discourse concerning the constitution of social order and society” (Stjerno, 2004, p. 30), especially in the context of the nineteenth century emergence of capitalism and the widespread social and political upheaval it caused. This gives the concept of solidarity strong ‘integrative’ overtones. One could argue that worldwide migration flows are today causing similar social and political turmoil, as indeed Zapata-Barrero is referring to in his paper when he points to how the “re-nationalization of policies, xenophobia, racism and intolerance are becoming a new “political ideology”” (p. 17). Again, the notion of solidarity is called upon to create cohesion and order in our twenty-first century superdiverse urban societies. This is clearly reflected in the papers of Zapata-Barrero and Modood, when they recognise the ‘limits’ of multiculturalism and the recognition of cultural differences. In this context, Zapata-Barrero argues for the need to adopt national civic integration policies to counter ‘boundless multiculturalism’ (p.5) and Modood claims that “minority accommodation is to take place in an ongoing historic nation-state, […] multiculturalizing it from the inside” (p.10).

Still, given our empirical observations, I would warn against stressing the integrative associations of the notion of solidarity in diversity too much for reasons of maintaining a normative or policy consensus on diversity management. We need to at least entertain the hypothesis that actually existing place-based solidarities in diversity may work against grounding them in the framework of nation-states and their tendencies towards cultural homogenisation. Modood’s understanding of multiculturalism seems more keenly aware of the relations of power underpinning the macro-level dialogue on diversity (see e.g. “the pre-dominance that the cultural majority entails in the shaping of national culture”, p.11, and his stress on making established institutions more inclusive), although Zapata-Barrero stresses the importance of ‘equal status contact’ and intercultural policies as an ‘anti-racist tool’ (p.8) but tends to situate this more on the micro-level of concrete interactions. One case study in which this became particularly clear focused on the sustained attempts of the youth movement Chiro to create more ethnic-cultural diversity in its local groups in Brussels (De Haene, Schuermans & Verschelden, 2014 - DieGem case study 3).^7^ Youth movements are a well-established and powerful model for generating solidarity by strengthening social bonds and individual and collective competencies through playing together. However, given its long history, the youth movement model is also strongly steeped in traditions, which may inadvertently turn away children and youngsters with a migration background (e.g. alcohol consumption, wearing of uniforms). One of the strategies of Chiro was to set up a separate Chiro group called BINT with Moroccan girls. Originally meant as a temporary project to ease the way to a ‘regular’ Chiro group, the girls turned it into in a transformative project by experimenting with and questioning the youth movement model. This led to a range of questions, for example is wearing a uniform essential for attending a Chiro activity (and can a headscarf be part of this) and is there no need to involve paid professionals to support local groups in superdiverse neighbourhoods? Especially the latter questions proved highly contentious as youth volunteers are at the core of the youth movement and are cautious to involve professionals.

## Conclusion

Both the arguments of Modood and Zapata-Barrero have much to recommend, respectively the importance of equal citizenship, making mainstream institutions more inclusive, the existence of groupings and the macro-level in Modood’s multiculturalism and the stress on the micro-level focus on proximity and practices, intercultural dialogue and equal status contact in Zapata-Barrero’s Interculturalism. Still, I have argued for a more sociological approach that sees living in diversity as a social fact to be analysed empirically. This requires an analytical framework that does not a priori privilege specific sources of solidarity such as shared norms and values and contact/encounter on normative-philosophical or policy grounds. To this end, I propose to focus on how people mobilise different sources of solidarity in their attempts to take shared responsibility for the concrete places where they live, work, learn and play together in superdiversity. I have subsequently shown that this micro-level focus does not mean that one ignores macro-level processes since the latter very much structure the places of everyday life in diversity. Finally, I have called for more attention to the transformative nature of solidarities in diversity.

## Footnotes

1 The research project is called DieGem – which stands for Diversity and Community Development – and was supported by IWT-Flanders under Grant [IWT/120022/SBO] between 2013 and 2016. I am grateful to the DieGem research team, in particular Joke Vandenabeele, Nick Schuermans, Maarten Loopmans and Thomas Swerts, for the joint development of the conceptual framework underlying the research project. More information can be found on the following website: http://www.solidariteitdiversiteit.be/diegem_en.php.

2 The full case stury report is available (only in Dutch) here: http://www.solidariteitdiversiteit.be/uploads/docs/cases/springdef.pdf

3 The full case stury report is available (only in Dutch) here: http://www.solidariteitdiversiteit.be/praktijkenbank.php?case=18

4 The full case study report is available (only in Dutch) here: http://www.solidariteitdiversiteit.be/uploads/docs/cases/rapport_leeggoed_vdef.pdf

5 The full case study report is available (only in Dutch) here: http://www.solidariteitdiversiteit.be/uploads/docs/cases/towerns07042016.pdf

6 The full case study report is available (only in Dutch) here: http://www.solidariteitdiversiteit.be/uploads/docs/cases/rapport_hivset.pdf

7 The full case study report is available (only in Dutch) here: http://www.solidariteitdiversiteit.be/uploads/docs/cases/rapportage_chiro_definitief.pdf
